# Heart disease in pregnancy and risk of pre-eclampsia: a Swedish register-based study

**DOI:** 10.1136/openhrt-2024-002728

**Published:** 2024-05-23

**Authors:** Karl Bergman, Teresia Svanvik, Carmen Basic, Annika Rosengren, Tatiana Zverkova Sandström, Jimmy Celind, Helen Sjöland, Anna-Karin Wikström, Maria Schaufelberger, Erik Thunström

**Affiliations:** 1 Department of Molecular and Clinical Medicine, Institute of Medicine, Sahlgrenska Academy, University of Gothenburg, Gothenburg, Sweden; 2 Division of Cardiology, Tygerberg Hospital, Stellenbosch University, Faculty of Medicine and Health Sciences, Cape Town, Western Cape, South Africa; 3 Department of Obstetrics and Gynecology, University of Gothenburg Institute of Clinical Sciences, Göteborg, Sweden; 4 Department of Pediatrics, University of Gothenburg Institute of Clinical Sciences, Göteborg, Sweden; 5 Sahlgrenska Osteoporosis Centre, University of Gothenburg Institute of Medicine, Göteborg, Sweden; 6 Department of Women's and Children's Health, Uppsala University, Uppsala, Sweden

**Keywords:** HEART FAILURE, Heart Valve Diseases, EPIDEMIOLOGY, Pregnancy

## Abstract

**Background and aims:**

Pre-eclampsia complicates 3–5% of pregnancies worldwide and is associated with adverse outcomes for the mother and the offspring. Pre-eclampsia and heart failure have common risk factors, including hypertension, obesity and diabetes. It is not known whether heart failure increases the risk of pre-eclampsia. This study examines whether pregestational heart failure increases the risk of pre-eclampsia.

**Methods:**

In a registry-based case–cohort study that included all pregnancies in Sweden (n=3 125 527) between 1990 and 2019, all pregnancies with pre-eclampsia (n=90 354) were identified and up to five control pregnancies (n=451 466) for each case were chosen, matched on the mother’s birth year. Multiple logistic regression analysis was used to evaluate the impact of heart failure on the risk of pre-eclampsia, with adjustment for established risk factors and other cardiovascular diseases.

**Results:**

Women with heart failure had no increased risk for pre-eclampsia, OR 1.02 (95% CI 0.69 to 1.50). Women with valvular heart disease had an increased OR of preterm pre-eclampsia, with an adjusted OR of 1.78 (95% CI 1.04 to 3.06). Hypertension and diabetes were independent risk factors for pre-eclampsia. Obesity, multifetal pregnancies, in vitro fertilisation, older age, Nordic origin and nulliparity were more common among women who developed pre-eclampsia compared with controls.

**Conclusion:**

Women with heart failure do not have an increased risk of pre-eclampsia. However, women with valvular heart disease prior to pregnancy have an increased risk of developing preterm pre-eclampsia independent of other known risk factors.

**WHAT IS ALREADY KNOWN ON THIS TOPIC?**

- Pre-eclampsia is a severe pregnancy complication associated with increased mortality and morbidity, both for the fetus and the mother. Women with a pregnancy complicated by pre-eclampsia have a higher risk for future cardiovascular disease, such as hypertension, ischaemic heart disease and heart failure. To what extent pregestational heart failure and other established cardiovascular diseases are associated with pre-eclampsia has not been as widely studied.

**WHAT THIS STUDY ADDS**

- Women with heart failure had no increased risk of pre-eclampsia. However, women with valvular heart disease showed an increased risk of preterm pre-eclampsia (≤37 weeks of gestation) and after adjusting for established risk factors of pre-eclampsia demonstrated an OR of 1.78 (95% CI 1.04 to 3.06). We could also confirm the association between other already known cardiovascular risk factors and pre-eclampsia, such as increased body mass index, diabetes and hypertension.

**HOW THIS STUDY MIGHT AFFECT RESEARCH, PRACTICE OR POLICY**

- There are already existing recommendations for women with heart failure to be offered preconception counselling and close surveillance during pregnancy, due to the risk of decompensation with potential harm for both the mother and the fetus. Our study confirms that women with pregestational heart failure do not have an increased risk of pre-eclampsia. Hence, women with heart failure do not routinely have to be offered low-dose aspirin as pre-eclampsia prophylaxis when being evaluated during pregnancy. Women with valvular heart disease have an increased risk for preterm pre-eclampsia. Therefore, these women should be considered for prophylaxis treatment for pre-eclampsia.

## Introduction

Pre-eclampsia accounts for approximately 42 000 maternal deaths annually, affecting 3–5% of all pregnancies worldwide.^1^ The most recent standard definition for pre-eclampsia is new-onset hypertension (≥140/90 mm Hg), with proteinuria or end organ dysfunction at any time after 20 weeks of gestation. It is considered a multiorgan disorder secondary to endothelial dysfunction.^1 2^

In a normotensive pregnancy there is a physiological adaptation with vasodilation, where the systemic vascular resistance decreases and cardiac output increases.^3^ In pre-eclampsia, by contrast, there is an altered haemodynamic profile with an inability to increase cardiac output and failure to induce the natural fall in systemic vascular resistance. This is most evident in early-onset pre-eclampsia (diagnosed before 34 weeks of gestation).^4^

Established risk factors for developing pre-eclampsia include previous pre-eclampsia, chronic hypertension, presence of antiphospholipid antibodies, systemic lupus erythematosus, rheumatoid arthritis, multiple pregnancy, advanced maternal age, renal disease, diabetes mellitus, high body mass index (BMI), nulliparity and in vitro fertilisation.^1 2^ To what extent heart failure, valvular heart disease and other established cardiovascular diseases are associated with pre-eclampsia has not been widely studied. There is some evidence that women with congenital heart disease have an increased risk of pre-eclampsia.^5^ However, the risk of pre-eclampsia in women with pregestational heart failure, a condition that is on the rise in the young, is unknown.^6^

The primary objective of this study was to determine the impact of pregestational heart failure on the risk of developing pre-eclampsia. The secondary objective was to determine whether pre-eclampsia has an earlier onset in individuals with pregestational heart failure.

## Methods

### Data sources and study cohort

This case–cohort study is a part of a larger epidemiological venture, SWED-PREG (SWEDish PREGnancy study), that conducts research using nationwide Swedish registries. The aim of the research project is to improve risk assessment in women with cardiovascular disease and their offspring during pregnancy, delivery and post partum. The study was carried out in accordance with the Declaration of Helsinki.^7^ The article is written according to the Strengthening the Reporting of Observational Studies in Epidemiology guidelines.^8^ The SWED-PREG project is registered in the National Research Database of Sweden at www.researchweb.org (project number: 277121).

Data for the study were derived from the nationwide Swedish Medical Birth Register, Longitudinal Integrated Database for Health Insurance and Labour Market Studies (LISA) and the National Patient Register, all with national coverage, and linked through the Swedish personal identification number (PIN), mandatory for all citizens in Sweden. Since the Swedish healthcare system is tax funded, this yields extremely high coverage in the registries.^9^

The Swedish Medical Birth Register was initiated in 1973 and covers over 98% of all pregnancies leading to deliveries in Sweden during the study period. It provides information about pregnancy, labour and perinatal outcome.^9^ The cases in the study are derived from all pregnancies with a concomitant diagnosis of pre-eclampsia, by International Classification of Diseases Ninth Revision (ICD-9) and ICD-10 codes, in the Medical Birth Register in the period 1990–2019. Data on pre-eclampsia diagnosis is considered to have good to very good validity in the Swedish Medical Birth Register.^9^ Validation of the diagnosis in the Norwegian Medical Birth Register, which holds great similarities with the Swedish registry, determined a high positive predictive value concerning diagnosis of pre-eclampsia.^10^

The controls were similarly derived from the Medical Birth Register, where five control pregnancies for each case without a pre-eclampsia diagnosis were sampled from the remaining pregnancies, matched on the mother’s year of birth. Since cases were defined by deliveries and not as individuals, the same woman could appear more than once in the analysis. In 303 of the cases, fewer than five controls were sampled since one, or for one case, two of the sampled controls died prior to index delivery. Therefore, the control group consisted of 451 466 deliveries. Pre-eclampsia, established risk factors for pre-eclampsia and comorbidities adjusted for in the statistical analysis are described in online supplemental table S2.

10.1136/openhrt-2024-002728.supp1 Supplementary data

**Figure 1:**
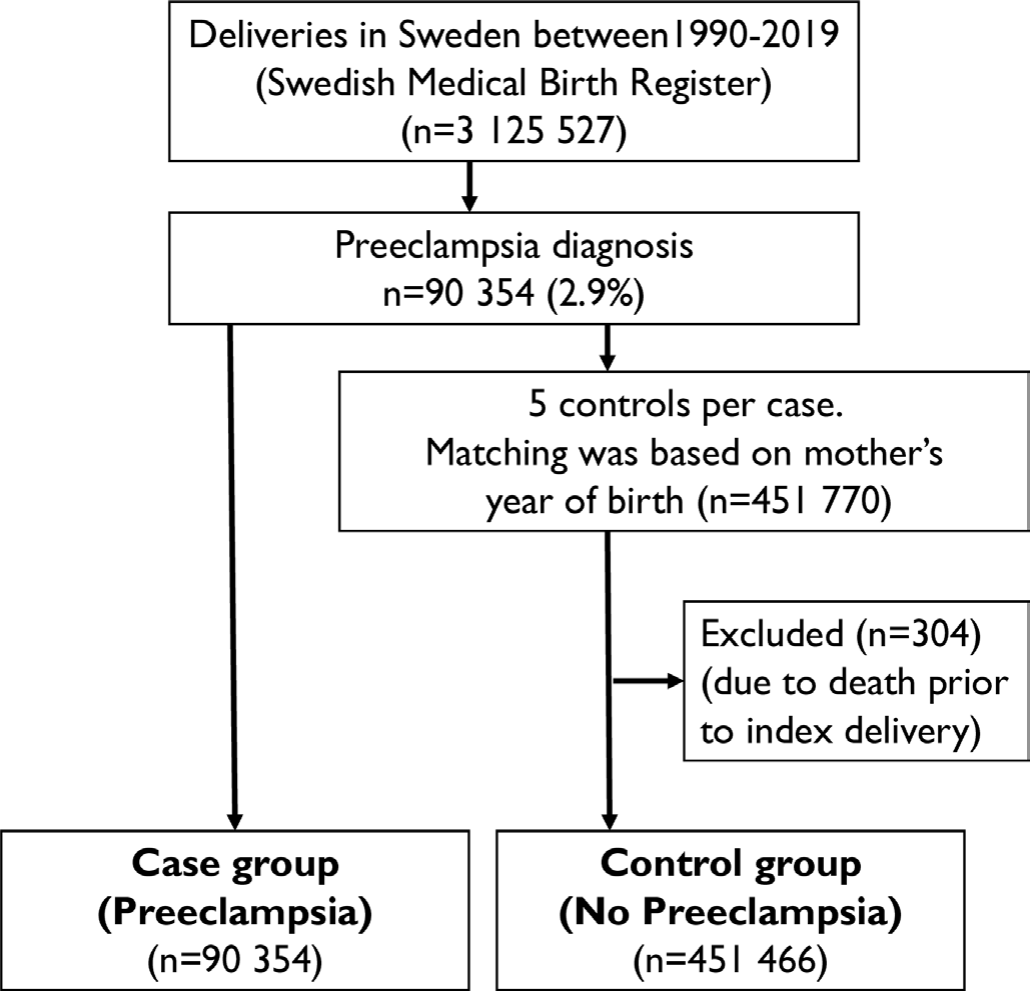
Inclusion and exclusion flow chart.

The case group was further subdivided into preterm and term pre-eclampsia. Preterm pre-eclampsia was defined as: (1) pre-eclampsia diagnosis and (2) delivery before the 37th week of gestation. Pre-eclampsia diagnoses and delivery in the 37th week of gestation or later were defined as term pre-eclampsia. Figure 1 shows the inclusion and exclusion flow chart.

From the Medical Birth Register, we derived data on maternal age, height, weight, BMI prior to pregnancy (using weight at the first antenatal visit registered since 1992 as a proxy for pre-pregnancy weight), gestational length at delivery and number of multifetal pregnancies. Diagnoses with respect to cardiovascular disease prior to the relevant pregnancy were collected from the National Patient Register, launched in 1964, and with nationwide coverage from 1987.^11^ The register includes inpatient and outpatient diagnoses and their ICD codes. The proportion of valid diagnoses in the National Patient Register is considered high in patients with cardiovascular disease.^12^ Comorbidities were included with ICD-9 and ICD-10 codes as diagnoses both in primary and contributory position. All women with a hypertension diagnosis entering the index pregnancy were classified as chronic hypertensives and only diagnosed with pre-eclampsia if they also developed proteinuria (superimposed pre-eclampsia). Cardiomyopathies were sorted together with heart failure; aortic disease and peripheral artery disease were combined into one group in the analysis, as were myocarditis and pericarditis (see table 1 and the full breakdown of diagnoses in online supplemental table S1).

**Table 1:** Population characteristics

	All pregnancies n (%)	Pre-eclampsia n (%)	Controls n (%)
Deliveries (n)	541 820	90 354	451 466
Age at delivery			
Mean (SD)	29.7 (5.3)	29.6 (5.5)	29.7 (5.2)
<20	10 311 (1.9)	2012 (2.2)	8299 (1.8)
20–24	83 559 (15.4)	14 960 (16.6)	68 599 (15.2)
25–29	176 198 (32.5)	28 976 (32.1)	147 222 (32.6)
30–34	170 279 (31.4)	26 634 (29.5)	143 645 (31.8)
35–39	82 448 (15.2)	13 837 (15.3)	68 611 (15.2)
40+	19 025 (3.5)	3935 (4.4)	15 090 (2.8)
Body mass index			
Mean (SD)	24.8 (4.7)	26.4 (5.5)	24.5 (4.5)
Missing or <15*	88 752 (16.4)	14 538 (16.1)	74 214 (16.4)
15.0 to <20.0	45 967 (8.5)	4898 (5.4)	41 069 (9.1)
20.0 to <22.5	118 087 (21.8)	14 475 (16.0)	103 612 (23.0)
22.5 to <25.0	115 639 (21.3)	17 271 (19.1)	98 368 (21.8)
25.0 to <27.5	72 291 (13.3)	13 013 (14.4)	59 278 (13.1)
<27.5 to <30.0	42 182 (7.8)	9008 (10.0)	33 174 (7.3)
30.0 to <35.0	40 845 (7.5)	10 817 (12.0)	30 028 (6.7)
35.0 to <40.0	13 263 (2.4)	4502 (5.0)	8761 (1.9)
40+	4794 (0.9)	1832 (2.0)	2962 (0.7)
Parity			
0	249 614 (46.2)	59 765 (66.2)	189 849 (42.1)
1	184 945 (34.1)	19 605 (21.7)	165 340 (36.6)
2	72 170 (13.3)	7333 (8.1)	64 837 (14.4)
3	21 897 (4.0)	2295 (2.5)	19 602 (4.3)
4 or more	13 194 (2.4)	1356 (1.5)	11 838 (2.6)
Origin			
Nordic countries	425 781 (78.6)	78 050 (86.4)	347 731 (77.0)
Rest of Europe	34 825 (6.4)	3828 (4.2)	30 997 (6.9)
Rest of the world	81 214 (15.0)	8476 (9.4)	72 738 (16.1)
Smoking	87 158 (16.1)	13 168 (14.6)	73 990 (16.4)
In vitro fertilisation	13 226 (2.5)	3 498 (3.9)	9768 (2.2)
Multifetal pregnancies	10 811 (2.0)	4842 (5.4)	5969 (1.3)
Comorbidities at baseline			
Congenital heart disease	1891 (0.35)	300 (0.33)	1591 (0.35)
Valvular heart disease	353 (0.07)	63 (0.07)	290 (0.06)
Arrhythmia	1296 (0.24)	183 (0.20)	1113 (0.25)
Hypertension	2040 (0.38)	1095 (1.21)	945 (0.21)
Heart failure	183 (0.03)	31 (0.03)	152 (0.03)
Myocarditis/pericarditis	165 (0.03)	29 (0.03)	136 (0.03)
Aortic disease/PAD	43 (0.01)	5 (0.01)	38 (0.01)
Myocardial infarction	65 (0.01)	13 (0.01)	52 (0.01)
Other coronary artery disease	62 (0.01)	6 (0.01)	56 (0.01)
Diabetes, type 1	3803 (0.70)	2105 (2.33)	1698 (0.38)
Diabetes, type 2	507 (0.09)	208 (0.23)	299 (0.07)
Rheumatoid arthritis	544 (0.10)	131 (0.14)	413 (0.09)
Systemic lupus erythematosus	547 (0.10)	179 (0.20)	368 (0.08)

*The Swedish Medical Birth Register has no data on body mass index (BMI) for 1990–1991. Observations with missing values are removed and not part of the calculations.

PAD, peripheral artery disease.

Data on country of origin and education were collected from Statistics Sweden’s LISA. The LISA Register comprises data on country of origin, sickness leave, parental insurance and unemployment insurance at the individual level. LISA enables the study of individuals’ transition over time between, for example, gainful employment, unemployment and illness. Currently, data are available from 1990 onwards for all persons aged 16 or older. From 2010 onwards, data on persons aged 15 years are also available.^13^

### Outcomes

Pre-eclampsia was the primary outcome. Swedish clinical guidelines defined pre-eclampsia during the study period as new-onset hypertension (blood pressure ≥140/90 mm Hg) combined with proteinuria (>0.3 g for 24 hours) at 20 weeks of gestation or later.

### Statistical analysis

Continuous variables are presented as means and SDs. Dichotomous and categorical data are presented as frequencies and percentages. ORs with 95% CI were calculated using logistic regression. All tests were two tailed and p<0.05 was considered significant. Pre-eclampsia during the study period (1990–2019) was considered the main outcome. Adjustments were performed for established risk factors for pre-eclampsia, according to the National Institute for Health and Care Excellence (NICE) guidelines.^14^ Model 1 is the univariate analysis; in model 2, adjustments for established risk factors were done; in model 3, adjustments for established risk factors and the cardiovascular disorders that were significant in the univariate analysis (model 1) were done; and in model 4, adjustments for established risk factors and all cardiovascular disorders were performed. To account for possible multicollinearity in the data, we used the generalised estimating equation under the assumption of binomial distribution of the response variable, which showed no significant difference in the results (online supplemental table S2). All statistical analyses and data management were performed using SAS V.9.4 (SAS Institute). Graphics were created in R V.4.2.2.

### Patient and public involvement

In this study, we had no patient nor public involvement.

## Results

**Figure 2:**
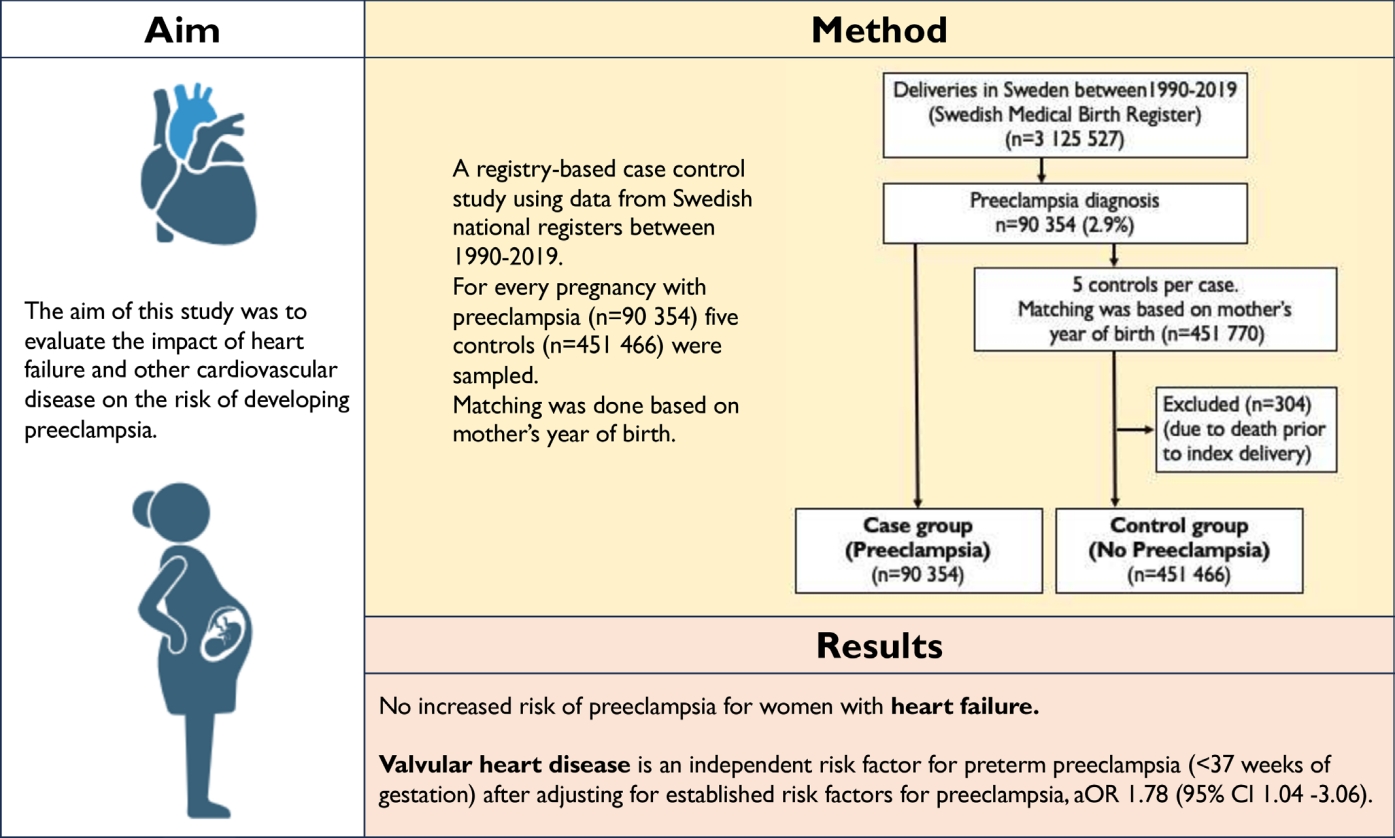
Visual summary. aOR, adjusted OR.

A total of 3 125 527 pregnancies were included in the cohort, and of those, pre-eclampsia was registered in 90 354 (2.9%). Out of the remaining pregnancies, up to five for each case, without a pre-eclampsia diagnosis, were sampled as controls (n=451 770), matched on the mother’s year of birth. Out of the 90 354 cases, 21 703 deliveries were preterm and 68 651 were term deliveries (figure 2) .

Baseline characteristics for pre-eclampsia and control pregnancies are presented in table 1. Mean BMI was higher in the pre-eclampsia group compared with the control group, with a markedly higher prevalence of obesity in the pre-eclampsia group (19% vs 9.3%). The majority of the women in the cohort, both in the pre-eclampsia group and the control group, were born in the Nordic countries. Nordic origin was more common for women with pre-eclampsia compared with women from the control group (86.4% vs 77.0%). Multifetal pregnancies were almost four times as common among women with pre-eclampsia compared with controls (5.4% vs 1.3%). The prevalence of cardiovascular disorders was generally low in both groups. However, hypertension and diabetes types 1 and 2 were more common in the pre-eclampsia group compared with the controls. Also, systemic lupus erythematosus and rheumatoid arthritis were both more common among the women with pre-eclampsia than controls.

Heart failure, valvular heart disease, congenital heart disease, myocardial infarction and other coronary heart disease were not associated with pre-eclampsia (table 2, model 1). Hypertension, rheumatoid arthritis, systemic lupus erythematosus and type 1 and type 2 diabetes were all statistically significantly associated with pre-eclampsia (table 2, model 1). Arrhythmia was associated with reduced risk for pre-eclampsia (table 2, model 1). In the univariable analysis for preterm pre-eclampsia, hypertension, rheumatoid arthritis, systemic lupus erythematosus, type 1 and type 2 diabetes and valvular heart disease were all statistically significantly associated with pre-eclampsia (table 3, model 1). Baseline heart failure, congenital heart disease, myocardial infarction, other coronary heart disease and arrhythmia were not associated with pre-eclampsia (table 3, model 1).

**Table 2:** Risk factors for pre-eclampsia

	OR (95% CI) Univariate—model 1	aOR (95% CI) Multivariate—model 2	aOR (95% CI) Multivariate—model 3	aOR (95% CI) Multivariate—model 4
Mother’s age				
<20	1.23 (1.17 to 1.30)	1.04 (0.99 to 1.10)	1.04 (0.98 to 1.09)	1.04 (0.99 to 1.09)
20–24	1.11 (1.08 to 1.13)	1.01 (0.98 to 1.03)	1.01 (0.98 to 1.03)	1.01 (0.98 to 1.03)
25–29	Reference	Reference	Reference	Reference
30–34	0.94 (0.93 to 0.96)	1.09 (1.07 to 1.11)	1.09 (1.07 to 1.11)	1.09 (1.07 to 1.11)
35–39	1.03 (1.02 to 1.05)	1.31 (1.28 to 1.34)	1.31 (1.28 to 1.34)	1.31 (1.28 to 1.34)
40+	1.33 (1.28 to 1.38)	1.68 (1.62 to 1.75)	1.69 (1.62 to 1.76)	1.69 (1.62 to 1.76)
Body mass index				
15 to <20	0.84 (0.83 to 0.88)	0.88 (0.85 to 0.91)	0.88 (0.85 to 0.92)	0.88 (0.85 to 0.92)
20 to <22.5	Reference	Reference	Reference	Reference
22.5 to <25	1.26 (1.23 to 1.29)	1.31 (1.28 to 1.35)	1.30 (1.27 to 1.33)	1.30 (1.27 to 1.33)
25 to <27.5	1.57 (1.53 to 1.61)	1.76 (1.71 to 1.81)	1.73 (1.68 to 1.78)	1.73 (1.68 to 1.78)
27.5 to <30	1.94 (1.89 to 2.00)	2.30 (2.23 to 2.37)	2.26 (2.19 to 2.33)	2.27 (2.20 to 2.33)
30 to <35	2.58 (2.51 to 2.65)	3.16 (3.07 to 3.25)	3.10 (3.01 to 3.20)	3.10 (3.01 to 3.20)
35 to <40	3.68 (3.53 to 3.83)	4.57 (4.39 to 4.77)	4.46 (4.28 to 4.65)	4.46 (4.28 to 4.65)
40+	4.43 (4.17 to 4.71)	5.51 (5.17 to 5.87)	5.38 (5.04 to 5.73)	5.38 (5.04 to 5.74)
Parity				
0	Reference			
1	0.38 (0.37 to 0.38)			
2	0.36 (0.35 to 0.37)			
3	0.37 (0.36 to 0.39)			
4 or more	0.36 (0.34 to 0.39)			
Origin				
Nordic countries	Reference	Reference	Reference	Reference
Rest of Europe	0.55 (0.53 to 0.60)	0.59 (0.57 to 0.61)	0.60 (0.57 to 0.62)	0.60 (0.57 to 0.62)
Rest of the world	0.52 (0.51 to 0.53)	0.53 (0.51 to 0.54)	0.53 (0.52 to 0.55)	0.53 (0.52 to 0.55)
Smoking	0.87 (0.85 to 0.89)	0.76 (0.74 to 0.77)	0.76 (0.57 to 0.62)	0.76 (0.74 to 0.77)
In vitro fertilisation	1.82 (1.75 to 1.89)	1.04 (1.00 to 1.09)	1.04 (1.00 to 1.09)	1.04 (1.00 to 1.09)
Nulliparity	2.69 (2.65 to 2.73)	3.06 (3.01 to 3.11)	3.07 (3.02 to 3.12)	3.07 (3.02 to 3.13)
Multifetal pregnancies	4.23 (4.07 to 4.39)	4.13 (3.96 to 4.30)	4.19 (4.02 to 4.37)	4.19 (4.02 to 4.37)
Comorbidities at baseline				
Congenital heart disease	0.94 (0.96 to 1.16)			1.05 (0.95 to 1.16)
Valvular heart disease	1.09 (0.83 to 1.43)			1.32 (0.95 to 1.85)
Arrhythmia	0.82 (0.70 to 0.96)			0.73 (0.62 to 0.86)
Hypertension	5.85 (5.36 to 6.38)		4.78 (4.35 to 5.26)	4.86 (4.41 to 5.35)
Heart failure	1.02 (0.69 to 1.50)			0.78 (0.51 to 1.19)
Myocarditis/pericarditis	1.07 (0.71 to 1.59)			0.89 (0.58 to 1.36)
Aortic disease/PAD	0.66 (0.26 to 1.67)			0.50 (0.19 to 1.35)
Myocardial infarction	1.25 (0.68 to 2.29)			0.57 (0.27 to 1.17)
Other coronary artery disease	0.54 (0.23 to 1.25)			0.49 (0.20 to 1.17)
Diabetes, type 1	6.31 (5.92 to 6.73)		5.23 (4.88 to 5.61)	5.24 (4.89 to 5.61)
Diabetes, type 2	3.49 (2.92 to 4.16)		2.40 (1.97 to 2.91)	2.41 (1.98 to 2.93)
Rheumatoid arthritis	1.59 (1.32 to 1.92)		1.26 (1.02 to 1.56)	1.26 (1.02 to 1.56)
Systemic lupus erythematosus	2.43 (2.04 to 2.91)		2.22 (1.83 to 2.69)	2.27 (1.87 to 2.76)

Model 1: univariate analysis. Model 2: adjusted for established risk factors (age, BMI, country/region of origin, in vitro fertilisation, parity, multifetal pregnancies). Model 3: adjusted for established risk factors and cardiovascular disorders that were significant in the univariate analysis. Model 4: adjusted for established risk factors and all cardiovascular disorders.

.aOR, adjusted OR; BMI, body mass index; PAD, peripheral artery disease.

**Table 3:** Risk factors for preterm pre-eclampsia

	OR (95% CI) Univariate—model 1	aOR (95% CI) Multivariate—model 2	aOR (95% CI) Multivariate—model 3	aOR (95% CI) Multivariate—model 4
Mother’s age				
<20	1.33 (1.20 to 1.47)	1.12 (1.01 to 1.24)	1.11 (1.00 to 1.24)	1.11 (1.00 to 1.24)
20–24	1.17 (1.12 to 1.22)	1.06 (1.01 to 1.12)	1.06 (1.01 to 1.12)	1.06 (1.01 to 1.12)
25–29	Reference	Reference	Reference	Reference
30–34	0.98 (0.95 to 1.02)	1.12 (1.08 to 1.17)	1.13 (1.08 to 1.17)	1.13 (1.08 to 1.17)
35–39	1.13 (1.08 to 1.18)	1.41 (1.34 to 1.48)	1.42 (1.35 to 1.49)	1.42 (1.35 to 1.49)
40+	1.37 (1.56 to 1.79)	2.00 (1.85 to 2.16)	2.02 (1.87 to 2.18)	2.02 (1.87 to 2.18)
Body mass index				
15 to <20	0.90 (0.84 to 0.96)	0.92 (0.86 to 0.99)	0.93 (0.87 to 1.00)	0.93 (0.87 to 1.00)
20 to <22.5	Reference	Reference	Reference	Reference
22.5 to <25	1.21 (1.15 to 1.27)	1.26 (1.20 to 1.32)	1.24 (1.18 to 1.30)	1.24 (1.18 to 1.30)
25 to <27.5	1.43 (1.36 to 1.51)	1.57 (1.49 to 1.66)	1.51 (1.43 to 1.60)	1.51 (1.43 to 1.60)
27.5 to <30	1.77 (1.67 to 1.88)	2.03 (1.90 to 2.16)	1.98 (1.86 to 2.11)	1.98 (1.86 to 2.11)
30 to <35	2.25 (2.12 to 2.38)	2.68 (2.52 to 2.85)	2.60 (2.44 to 2.77)	2.60 (2.44 to 2.77)
35 to <40	3.23 (2.97 to 3.52)	3.98 (3.64 to 4.36)	3.81 (3.48 to 4.18)	3.82 (3.49 to 4.18)
40+	4.14 (3.65 to 4.69)	5.08 (4.45 to 5.81)	4.87 (4.25 to 5.57)	4.87 (4.25 to 5.58)
Parity				
0	Reference			
1	0.36 (0.35 to 0.38)			
2	0.39 (0.37 to 0.41)			
3	0.41 (0.38 to 0.45)			
4 or more	0.39 (0.35 to 0.44)			
Origin				
Nordic countries	Reference	Reference	Reference	Reference
Rest of Europe	0.61 (0.57 to 0.66)	0.65 (0.61 to 0.70)	0.67 (0.62 to 0.72)	0.67 (0.62 to 0.72)
Rest of the world	0.70 (0.67 to 0.73)	0.73 (0.70 to 0.76)	0.76 (0.72 to 0.79)	0.76 (0.72 to 0.79)
Smoking	0.81 (0.77 to 0.84)	0.74 (0.71 to 0.77)	0.73 (0.70 to 0.76)	0.73 (0.70 to 0.76)
In vitro fertilisation	2.32 (2.15 to 2.50)	1.14 (1.05 to 1.24)	1.14 (1.04 to 1.24)	1.14 (1.04 to 1.24)
Nulliparity	2.67 (2.59 to 2.75)	2.99 (2.89 to 3.09)	3.03 (2.93 to 3.14)	3.03 (2.93 to 3.14)
Multifetal pregnancies	9.27 (8.67 to 9.91)	8.62 (8.03 to 9.25)	9.00 (8.38 to 9.66)	8.95 (8.34 to 9.61)
Comorbidities at baseline				
Congenital heart disease	0.94 (0.74 to 1.20)			1.17 (0.96 to 1.42)
Valvular heart disease	1.86 (1.15 to 4.47)	1.98 (1.18 to 3.33)	1.78 (1.04 to 3.06)	1.73 (1.00 to 3.01)
Arrhythmia	0.84 (0.61 to 1.14)			0.70 (0.49 to 0.98)
Hypertension	10.08 (8.61 to 11.80)		8.59 (7.23 to 10.21)	8.77 (7.37 to 10.44)
Heart failure	1.71 (0.91 to 3.22)	1.29 (0.65 to 2.55)	0.73 (0.35 to 1.53)	0.87 (0.37 to 1.63)
Myocarditis/pericarditis	1.48 (0.67 to 3.26)			1.14 (0.47 to 2.75)
Aortic disease/PAD	1.00 (0.22 to 4.56)			0.41 (0.07 to 2.32)
Myocardial infarction	2.52 (0.86 to 7.37)			1.02 (0.23 to 4.64)
Other coronary artery disease	0.77 (0.17 to 3.41)			0.64 (0.13 to 3.17)
Diabetes, type 1	11.71 (10.39 to 13.20)		10.82 (9.52 to 12.29)	10.83 (9.53 to 12.30)
Diabetes, type 2	4.19 (3.03 to 5.80)		2.52 (1.74 to 3.65)	2.53 (1.74 to 3.66)
Rheumatoid arthritis	2.36 (1.64 to 3.40)		1.83 (1.22 to 2.75)	1.83 (1.21 to 2.75)
Systemic lupus erythematosus	5.01 (3.71 to 6.77)		4.83 (3.47 to 6.73)	5.12 (3.67 to 7.14)

Model 1: univariate analysis. Model 2: adjusted for established risk factors (age, BMI, country/region of origin, in vitro fertilisation, parity, multifetal pregnancies). Model 3: adjusted for established risk factors and cardiovascular disorders that were significant in the univariate analysis. Model 4: adjusted for established risk factors and all cardiovascular disorders.

aOR, adjusted OR; BMI, body mass index; PAD, peripheral artery disease.

Both younger and older mothers had an increased odds of pre-eclampsia compared with a reference group of women aged 25–29 years. Multifetal pregnancies, in vitro fertilisation, older age, higher BMI and nulliparity were more common among women with pre-eclampsia.

**Figure 3:**
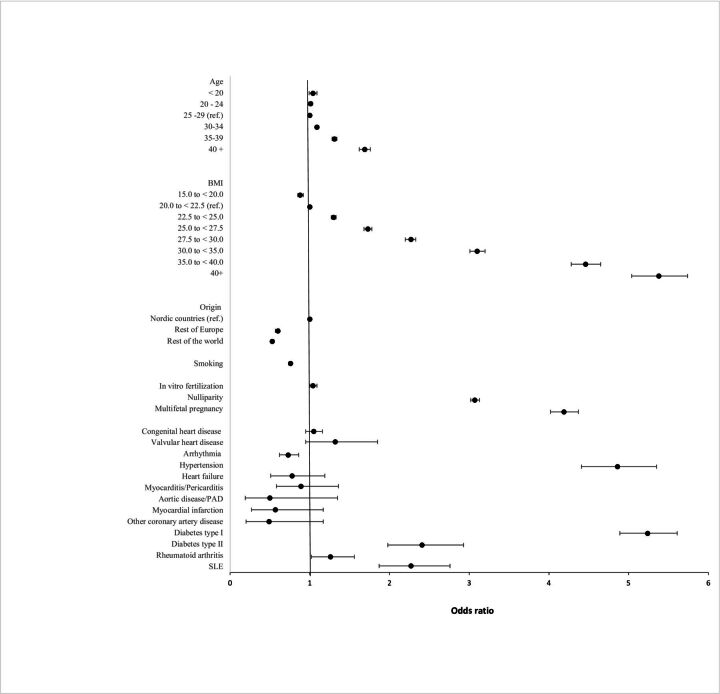
Forest plot of risk factors for pre-eclampsia. Adjusted for *age,* body mass index (BMI), country/region of origin, smoking, in vitro fertilisation, nulliparity, multifetal pregnancies, rheumatoid arthritis, and systemic lupus erythematosus (SLE) and all cardiovascular disorders. PAD, peripheral artery disease.

In the final multivariable model (model 4), higher age, starting at age 30 and older, higher BMI, starting at high normal, or BMI≥22.5, and with obesity class I, II and III associated with threefold, fourfold and fivefold increase in odds of pre-eclampsia compared with the low-normal reference group (BMI 20 to <22.5), nulliparity and multifetal pregnancies were all associated with increased risk. Smoking reduced the risk of pre-eclampsia. Of the comorbidities, both types of diabetes, systemic lupus erythematosus and rheumatoid arthritis were associated with pre-eclampsia. Arrhythmia was associated with reduced risk for pre-eclampsia (figure 3).

Valvular heart disease had an association with preterm pre-eclampsia. In the univariable analysis, the OR for valvular heart disease was 1.86 (95% CI 1.15 to 4.47). After adjusting for established risk factors (age, BMI, country/region of origin, in vitro fertilisation, parity and multifetal pregnancies), valvular heart disease was significantly associated with increased odds for preterm pre-eclampsia (table 3, model 2).

**Figure 4:**
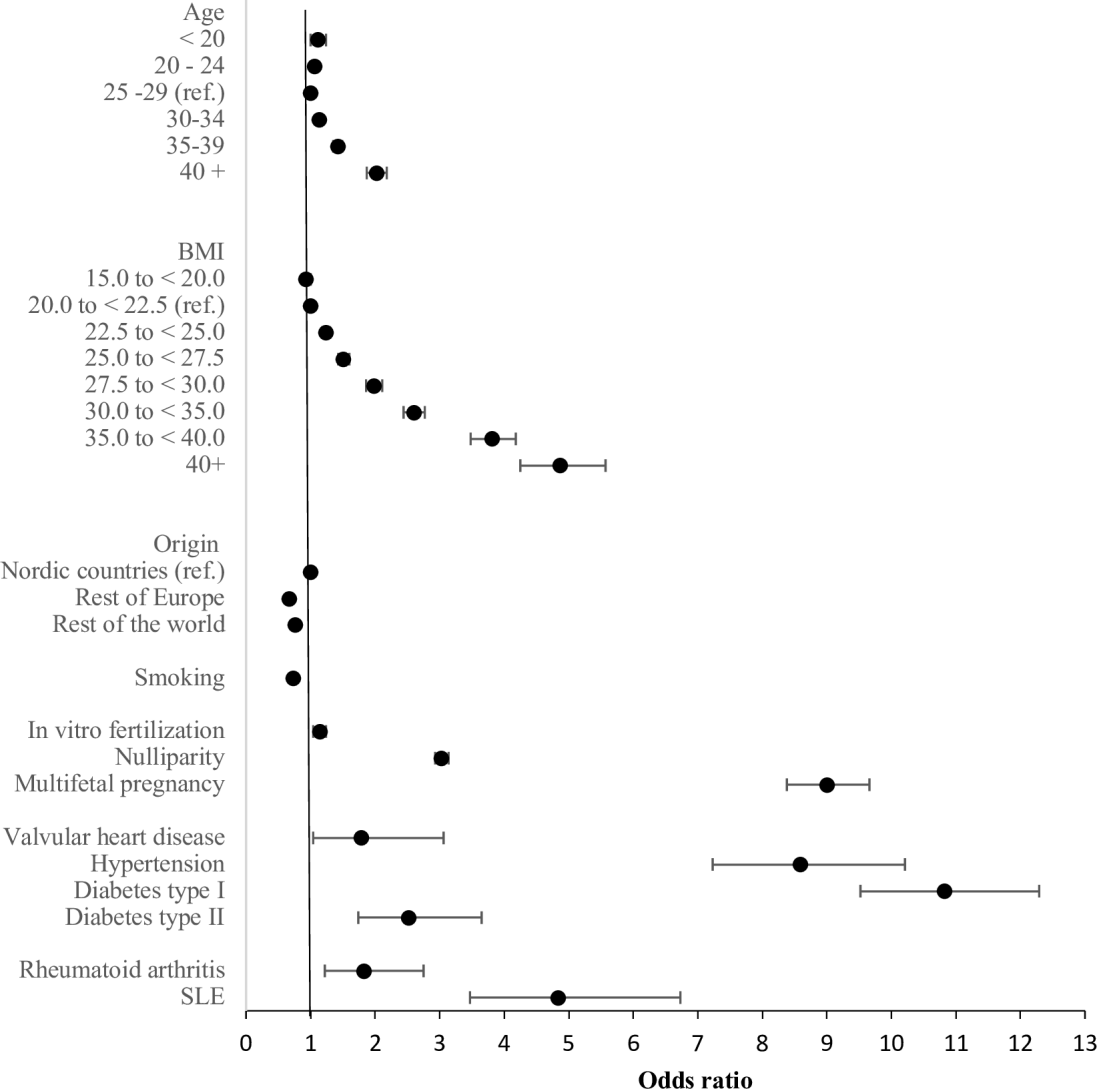
Forest plot of risk factors for preterm pre-eclampsia. Adjusted for age, body mass index (BMI), country/region of origin, smoking, in vitro fertilisation, nulliparity, multifetal pregnancies, valvular heart disease, hypertension, diabetes, rheumatoid arthritis and systemic lupus erythematosus (SLE).

Valvular heart disease remained associated with preterm pre-eclampsia after adding systemic lupus erythematosus, rheumatoid arthritis and all cardiovascular disorders that were significant in the univariable analysis to the logistical model (table 3, model 3). The adjusted OR for valvular heart disease was 1.78 (95% CI 1.04 to 3.06) (figure 4). This association with preterm pre-eclampsia persisted after adding all registered cardiovascular disorders independent of significance in the univariate model (table 3, model 4).

## Discussion

We found that being registered with a heart failure or cardiomyopathy diagnosis prior to pregnancy was not associated with an increased risk of pre-eclampsia. However, our study confirmed prior studies showing older maternal age, any BMI higher than low normal, nulliparity, multifetal pregnancy, type 1 and 2 diabetes, rheumatoid arthritis and systemic lupus erythematosus were associated with an increased risk of developing pre-eclampsia, and that smoking was associated with a decreased risk. We also found an independent association between valvular heart disease and preterm pre-eclampsia.

Among all women in Sweden who were pregnant and were delivered during a 30-year period, only 183 had been diagnosed with heart failure before the index pregnancy.

There is an increased risk for women with a pregnancy complicated by pre-eclampsia to develop heart failure later in life.^15^ We hypothesised an association between pregestational heart failure and pre-eclampsia, which we could not confirm. During a normal pregnancy, cardiac output increases and the systemic vascular resistance and blood pressure decrease.^3^ Foo *et al* 16 have shown that women who later develop pre-eclampsia (independent of prior heart failure) have lower cardiac output, higher systemic vascular resistance and higher blood pressure before pregnancy.^16^ Women with heart failure prior to pregnancy also have lower cardiac output and higher peripheral resistance.^17^ Increased low-grade inflammation is another common pathway that could result in both heart failure and pre-eclampsia. It is well established that prolonged inflammation might lead to heart failure.^18^ Pre-eclampsia is a proinflammatory state and women with pre-existing endothelial dysfunction are more susceptible to developing pre-eclampsia.^1^ It has also been reported that women with pre-pregnancy hypertension, diabetes or obesity have an increased risk of developing pre-eclampsia.^1^ All of these conditions also increase the risk of heart failure.^19^ Even though this could mean that women with heart failure would be less able to tolerate the cardiovascular adjustments that take place during pregnancy, we could not find that these women were more susceptible to pre-eclampsia.

We found an association between valvular heart disease and preterm pre-eclampsia; valvular heart disease remained a risk factor for preterm pre-eclampsia even when adjusted for hypertension, diabetes and obesity. Valvular heart disease is not an established risk factor for pre-eclampsia. Early delivery among individuals with preterm pre-eclampsia decreases the risk of complications both for the mother and for the fetus.^20^ Valvular heart disease could be a trigger for preterm pre-eclampsia since it increases the risk for reduced cardiac output with a disturbed placental circulation as consequence. There are data supporting that valvular heart disease is a risk factor for all forms of pre-eclampsia (preterm and term pre-eclampsia), a finding we could not confirm.^21^

To our knowledge, no previous studies have investigated the association between heart failure prior to pregnancy and risk of pre-eclampsia. Heart failure is a rare diagnosis among fertile women, which makes it hard to identify an adequate number of individuals prior to pregnancy; even with our large dataset we found less than 200 women with this condition.

Hayward *et al* 5 found an association between adult congenital heart disease and increased risk of future pre-eclampsia,^5^ but such an association was not found in our cohort. Since all pregnant women with congenital heart disease in Sweden were included in our study population, the lack of an association between congenital heart disease and risk of developing pre-eclampsia might be explained by the relative low number of cases of complex congenital heart disease, where haemodynamic status is more compromised than in non-complex disease. Women with a milder form of congenital heart disease are more likely to tolerate the haemodynamic changes taking place during pregnancy.

High BMI is a well-known risk factor for pre-eclampsia, with any pre-pregnancy BMI higher than 22.5 kg/m^2^ associated with higher odds of gestational hypertensive disorders compared with lower BMI.^22^ While the NICE guidelines^14^ indicate a BMI of ≥35 kg/m^2^ as moderate risk, the odds that we found in this study was more than fourfold, with a doubling of the odds in the overweight category, which represented a large proportion of the women in our study. A high BMI is a risk factor for heart failure,^23^ and cardiomyopathy^24^ in younger women, and also a risk factor for valvular heart disease.^25^ Accordingly, maintaining a healthy body weight is an important preventive measure for several reasons.

### Strength and limitations

A strength of this study is the large population-based dataset of more than 3.1 million deliveries, including information on the main risk factors for pre-eclampsia, and that almost all pregnancies in the whole of Sweden were included; thus, the risk of inclusion bias is low. Since every Swedish citizen has a PIN, we could also cross-reference different registries with each other. These registries have existed for many years with near-complete national coverage and with validated diagnoses for the conditions relevant for the present study,^9 12 13^ providing an opportunity for an extended inclusion and follow-up period which is needed to evaluate the association between disorders that are uncommon or rare among younger women, such as heart failure and pre-eclampsia.

However, there are also several limitations in this study. One is that we were restricted to the variables within our registries. On the other hand, having a large amount of data which is well validated makes it feasible to conduct high-quality research. Another limitation is that we do not know in detail in all cases what kind of heart failure or valvular heart disease the women in the cohort were suffering from since the diagnoses are based on ICD codes. Furthermore, no echocardiography data were available with details about the severity of valvular disease or data on ejection fraction in women with heart failure.

The established risk factors for pre-eclampsia we included in this study were chosen in accordance with the NICE guidelines.^14^ One limitation is that we did not include renal failure or antiphospholipid syndrome in this study because these parameters are poorly documented in our registries. The prevalence of these conditions was also extremely low, which is why under-reporting seemed likely. Therefore, we excluded these risk factors from this study.

### Generalisability

These results should be interpreted with caution in a global perspective, since black women who develop pre-eclampsia have higher rates of short-term and long-term morbidity and mortality than Caucasian women, and Swedish women are to a large extent Caucasian.^26^ For western countries with similar healthcare systems, our results are generalisable since this study covers almost all pregnant (98%) women between 1990 and 2019 in Sweden. It is a large dataset, which gives us the opportunity to examine a potential association between cardiac conditions that are rare in young women and the risk of future pre-eclampsia. We were able to confirm an association between previously known risk factors and pre-eclampsia, which underscores the validity and generalisability of our results.

## Conclusion

We found no increased risk for women with pregestational heart failure to develop pre-eclampsia. However, in a subgroup analysis, we found an association between valvular heart disease and preterm pre-eclampsia. Both heart failure and valvular heart disease are uncommon among women of fertile age in Sweden . Future research on other pregnancy complications for women with pregestational cardiovascular disease should be encouraged with the aim to reduce maternal morbidity and mortality.

## Data Availability

Data are available upon reasonable request. Data are available from the sources stated in the paper on request to the data providers, fulfilling legal and regulatory requirements and with permission from the Swedish Ethical Review Authority.
