# Supplementary material for: Heart disease in pregnancy and risk of pre-eclampsia: a Swedish register-based study
Source: Open Heart. 2024 May 23;11(1):e002728. doi: 10.1136/openhrt-2024-002728 (PMC11116857; doi:10.1136/openhrt-2024-002728)
Supplement: Supplementary data [file openhrt-2024-002728supp001.pdf]

Supplement 1

Table S1. ICD codes for each diagnosis

**Diagnosis**	**ICD-10**	**ICD-9**	**Data source**
**Preeclampsia**	O140, O141, O141A, O141B, O141X, O149, O119	642E, 642F, 642G, 642H, (6424, 6425)	MFR
**Congenital Heart Disease**			
Common arterial trunk	Q200	7450	NPR
Transposition of the great vessels	Q203	7451	NPR
Tetralogy of Fallot	Q213	7452	NPR
Ventricular septal defect	Q210	7450, 7454, 7468	NPR
Atrial septal defect or patent foramen ovale	Q211	7455, 7456, 7474	NPR
Congenital tricuspid stenosis or atresia	Q224	7461, 7468	NPR
Ebstein's anomaly	Q225	7462, 7468	NPR
Congenital stenosis of the aortic valve	Q230	7463, 7468	NPR
Congenital insufficiency of the aortic valve	Q231	7464, 7468	NPR
Congenital mitral stenosis	Q232	7465, 7468	NPR
Congenital mitral insufficiency	Q233	7466	NPR
Hypoplastic left heart syndrome	Q234	7467	NPR
Congenital subaortic stenosis	Q244	7468	NPR
Cor triatriatum	Q242	7468	NPR
Infundibular pulmonic stenosis	Q243	7468	NPR
Congenital coronary vessel anomalies	Q245	7468, 7469	NPR
Congenital heart block	Q246	7468	NPR

Coarctation of the aorta	Q251	7471	NPR
Atresia of aorta	Q252	7471, 7472	NPR
Stenosis of aorta	Q253	7472	NPR
Other congenital malformations of aorta	Q254	7472	NPR
Other congenital malformations of great arteries	Q258	7478	NPR
Congenital malformation of great arteries, unspecified	Q259	7478	NPR
Atresia of pulmonary artery	Q255	7473	NPR
Stenosis of pulmonary artery	Q256	7473	NPR
Other congenital malformations of pulmonary artery	Q257	7473	NPR
Congenital stenosis of vena cava	Q260	7474	NPR
Persistent left superior vena cava	Q261	7474	NPR
Total anomalous pulmonary venous connection	Q262	7474	NPR
Partial anomalous pulmonary venous connection	Q263	7474	NPR
Anomalous pulmonary venous connection unspecified	Q264	7474	NPR
Cor biloculare	Q208	7457, 7468, 7469	NPR
Double outlet right ventricle	Q201	7451	NPR
Double outlet left ventricle	Q202	7451	NPR
Double inlet ventricle	Q204	7453, 7468	NPR
Discordant atrioventricular connection	Q205	7451, 7454, 7468	NPR
Isomerism of atrial appendages	Q206	7468	NPR
Other congenital malformations of cardiac chambers and connections	Q208	7457, 7468, 7469	NPR
Congenital malformation of cardiac chambers and connections, unspecified	Q209	7468, 7469	NPR
Atrioventricular septal defects	Q212	7454, 7456	NPR
Aortopulmonary septal defect	Q214	7450	NPR
Other congenital malformations of the cardiac septum	Q218	7452, 7454, 7458	NPR

Unspecified congenital malformations of the cardiac septum	Q219	7459	NPR
Pulmonary valve atresia	Q220	7460	NPR
Congenital stenosis of the pulmonary valve	Q221	7460	NPR
Congenital pulmonary valve insufficiency	Q222	7460	NPR
Other congenital malformations of the pulmonary valve	Q223	7460	NPR
Hypoplastic right heart syndrome	Q226	7468	NPR
Other congenital malformations of the tricuspid valve	Q228	7468	NPR
Other congenital malformations of aortic and mitral valves	Q238	7468, 7469	NPR
Congenital malformation of aortic and mitral valves, unspecified	Q239	7469	NPR
Other specified congenital malformations of the heart	Q248	7449, 7468, 7469	NPR
Unspecified congenital malformations of the heart	Q249	7469	NPR
Patent ductus arteriosus	Q250	7470	NPR
**Pulmonary hypertension**			
Primary pulmonary hypertension	I270	4160	NPR
Other secondary pulmonary hypertension	I272	-	NPR
**Aortic disease**			
Thoracic aortic aneurysm, ruptured	I711	4411, 4415	NPR
Thoracic aortic aneurysm, without mention of rupture	I712	4412, 4416	NPR
Abdominal aortic aneurysm, ruptured	I713	4413	NPR
Abdominal aortic aneurysm, without mention of rupture	I714	4414	NPR
Thoracoabdominal aortic aneurysm, ruptured	I715	4415	NPR
Thoracoabdominal aortic aneurysm, without mention of rupture	I716	4416	NPR
Aortic aneurysm of unspecified site, ruptured	I718	4415	NPR

Aortic aneurysm of unspecified site, without mention of rupture	I719	4416	NPR
Dissection of aorta	I710	4410	NPR
**Peripheral artery disease**			
Claudicatio intermittens	I739B	4439	NPR
Aneurysm and dissection of carotid artery	I720	4428	NPR
Aneurysm and dissection of artery of upper extremity	I721	4420	NPR
Aneurysm of renal artery	I722A	4421	NPR
Dissection of renal artery	I722B	4421	NPR
Aneurysm and dissection of iliac artery	I723	4422	NPR
Aneurysm and dissection of artery of lower extremity	I724	4423	NPR
Aneurysm and dissection of other precerebral arteries	I725	-	NPR
Aneurysm and dissection of vertebral artery	I726	-	NPR
Aneurysm and dissection of other specified arteries	I728	4428	NPR
Aneurysm and dissection of unspecified site	I729	4210	NPR
Valvular heart disease			
Mitral stenosis	I050	3940	NPR
Nonrheumatic mitral (valve) stenosis	I342	4240	NPR
Mitral stenosis with mitral insufficiency	I052	3942	NPR
Rheumatic mitral insufficiency	I051	3941	NPR
Mitral (valve) insufficiency	I340	4240	NPR
Mitral (valve) prolapse	I341	4240	NPR
Rheumatic aortic stenosis	I060	3950	NPR
Aortic (valve) stenosis	I350	4241	NPR
Rheumatic aortic stenosis with insufficiency	I062	3952	NPR
Aortic (valve) stenosis with insufficiency	I352	4241	NPR
Rheumatic aortic insufficiency	I061	3951	NPR
Aortic (valve) insufficiency	I351	4241	NPR

Tricuspid insufficiency	I071	3970	NPR
Nonrheumatic tricuspid (valve) insufficiency	I361	4242	NPR
Pulmonary valve stenosis	I370	4243	NPR
Disorders of both mitral and aortic valves	I080	396	NPR
Disorders of both mitral and tricuspid valves	I081	3989	NPR
Disorders of both aortic and tricuspid valves	I082	3989	NPR
Combined disorders of mitral, aortic and tricuspid valves	I083	3989	NPR
Other multiple valve diseases	I088	3989	NPR
Multiple valve disease, unspecified	I089	3949	NPR
Mechanical valve prostheses	Z952	V433	NPR
Bioprostheses	Z953	V422	NPR
**Myocardial infarction**			
Unstable angina	I200	411	NPR
Acute transmural myocardial infarction of anterior wall	I210	410	NPR
Acute transmural myocardial infarction of inferior wall	I211	410	NPR
Acute transmural myocardial infarction of other sites	I212	410	NPR
Acute transmural myocardial infarction of unspecified site	I213	410	NPR
Acute subendocardial myocardial infarction	I214	410	NPR
Acute myocardial infarction, unspecified	I219	410	NPR
Subsequent myocardial infarction of anterior wall	I220	410	NPR
Subsequent myocardial infarction of inferior wall	I221	410	NPR
Subsequent myocardial infarction of other sites	I228	410	NPR
Subsequent myocardial infarction of unspecified site	I229	410	NPR
**Other coronary artery disease**			
Angina pectoris with documented spasm	I201	413	NPR

Other forms of angina pectoris	I208	413	NPR
Angina pectoris, unspecified	I209	413	NPR
Coronary artery aneurysm and dissection	I254	4141	NPR
**Heart failure**			
Peripartum cardiomyopathy	O903	6748	NPR
Dilated cardiomyopathy	I420	4254	NPR
Obstructive hypertrophic cardiomyopathy	I421	4251	NPR
Other hypertrophic cardiomyopathy	I422	4254	NPR
Endomyocardial (eosinophilic) disease	I423	4210	NPR
Endocardial fibroelastosis	I424	4253	NPR
Other restrictive cardiomyopathy	I425	4254	NPR
Alcoholic cardiomyopathy	I426	4255	NPR
Cardiomyopathy due to drugs and other external agents	I427	4259	NPR
Other cardiomyopathies	I428	4252, 4254	NPR
Cardiomyopathy, unspecified	I429	4254, 4259	NPR
Congestive heart failure	I500	4280	NPR
Cardiogenic shock	R570	7855	NPR
Left ventricular failure with reduced EF	I501A	4281	NPR
Left ventricular failure with slight impairment	I501B	4281	NPR
Left ventricular failure with preserved EF	I501C	4281	NPR
Left ventricular failure, unspecified	I501X	4281	NPR
Heart failure, unspecified	I509	4299	NPR
**Arrythmia**			
Ventricular tachycardia	I472	4271	NPR
Ventricular fibrillation	I490	4274	NPR
Supraventricular tachycardia	I471	4270, 4278	NPR
Wolff-Parkinson-White syndrome	I456A	4267, 4268	NPR
Lown-Ganong-Levine syndrome	I456B	4267, 4268	NPR
Pre-excitation syndrome, other type	I456W	4267, 4268	NPR
Pre-excitation syndrome	I456X	4267, 4268	NPR

Paroxysmal atrial fibrillation	I480	4273	NPR
Persistent atrial fibrillation	I481	4273	NPR
Chronic atrial fibrillation	I482	4273	NPR
Typical atrial flutter	I483	4273	NPR
Atypical atrial flutter	I484	4273	NPR
Atrial fibrillation and atrial flutter, unspecified	I489	4273	NPR
Nodal rhythm disorder	I498C	4260, 4278	NPR
Brugada syndrome	I498D	4260, 4278	NPR
Long QT syndrome	I498E	4260, 4278	NPR
Other specified cardiac arrythmias	I498W	4260, 4278	NPR
Sinus arrest	I495A	4278	NPR
Sick sinus syndrome	I495B	4278	NPR
Tachycardia-bradycardia syndrome	I495C	4278	NPR
Atrioventricular block, first degree	I440	4261	NPR
Atrioventricular block, Wenckebach block	I441A	4261	NPR
Atrioventricular block, Mobitz type II	I441B	4261	NPR
Atrioventricular block, complete	I442	4260	NPR
Other and unspecified atrioventricular block	I443	4261	NPR
Left anterior fascicular block	I444	4262	NPR
Left posterior fascicular block	I445	4262	NPR
Left bundle-branch block	I446A	4262	NPR
Left bundle-branch hemiblock	I446B	4262	NPR
Left bundle-branch block, unspecified	I447	4263	NPR
Right fascicular block	I450A	4264	NPR
Right fascicular hemiblock	I450B	4264	NPR
Other and unspecified right bundle-branch block	I451	4264	NPR
Bifascicular block	I452	4265, 4266	NPR
Trifascicular block	I453	4265	NPR
Nonspecific intraventricular block	I454	4265, 4266	NPR
Other specified heart block	I455	4261, 4266	NPR
Other specified conduction disorders	I458	4268, 4269	NPR

Conduction disorder, unspecified	I459	4266	NPR
Cardiac arrest with successful resuscitation	I460	4275	NPR
Sudden cardiac death, so described	I461	4299	NPR
Cardiac arrest, unspecified	I469	4275, 4289	NPR
**Hypertension**			
Essential (primary) hypertension	I109	4010, 4011, 4019	NPR
Hypertensive heart disease with (congestive) heart failure	I110	4020, 4021, 4029	NPR
I11.9 Hypertensive heart disease without (congestive) heart failure	I119	4020, 4021, 4029	NPR
Hypertensive renal disease with renal failure	I120	4030, 4031, 4039	NPR
Hypertensive renal disease without renal failure	I129	4039	NPR
Hypertensive heart and renal disease with (congestive) heart failure	I130	4040, 4041, 4049	NPR
Hypertensive heart and renal disease with renal failure	I131	4040, 4041, 4049	NPR
Hypertensive heart and renal disease with both (congestive) heart failure and renal failure	I132	4040, 4041, 4049	NPR
Hypertensive heart and renal disease, unspecified	I139	4040, 4041, 4049	NPR
Renovascular hypertension	I150	4050, 4051, 4059	NPR
Hypertension secondary to other renal disorders	I151	4050, 4051, 4059	NPR
Hypertension secondary to endocrine disorders	I152	4050, 4051, 4059	NPR
Other secondary hypertension	I158	4050, 4051, 4059	NPR
Secondary hypertension, unspecified	I159	4050, 4051, 4059	NPR
**Pericarditis / myocarditis**			
Acute nonspecific idiopathic pericarditis	I300	4209	NPR
Infective pericarditis with cardiac tamponade	I301A	4209	NPR
Infective pericarditis without cardiac tamponade	I301B	4209	NPR
Other forms of acute pericarditis with cardiac tamponade	I308A	4209	NPR

Other forms of acute pericarditis without cardiac tamponade	I308B	4209	NPR
Acute pericarditis, unspecified with cardiac tamponade	I309A	4209	NPR
Acute pericarditis, unspecified without cardiac tamponade	I309B	4209	NPR
Chronic adhesive pericarditis	I310	4231	NPR
Chronic constrictive pericarditis	I311	4232	NPR
Infective myocarditis	I400	4229	NPR
Isolated myocarditis	I401	4229	NPR
Other acute myocarditis	I408	4229	NPR
Acute myocarditis, unspecified	I409	4229	NPR
Myocarditis in bacterial diseases classified elsewhere	I410	4220A	NPR
Myocarditis in viral diseases classified elsewhere	I411	4220A, 4290	NPR
Myocarditis in other infectious and parasitic diseases classified elsewhere	I412	4220A, 4256A	NPR
Myocarditis in other diseases classified elsewhere	I418	4290	NPR
**Other**			
Diabetes, type 1	E10	250	NPR
Diabetes, type 2	E11	250	NPR
Rheumatoid arthritis	M0503, M0508, M0509, M060, M068, M069	7140, 7148	NPR
Systemic lupus erythematosus	M321, M328, M329	7100	MFR

(ICD = International Classification of Diseases. MFR= Medical Birth Register, NPR= National Patient Register.)

Table S2. Risk factors for preeclampsia.

	OR (95% CI) Any preeclampsia	aOR (95% CI) Preterm preeclampsia	aOR (95% CI) Term preeclampsia
**Mother's age**			
<20	1.48 (1.39-1.57)	1.57 (1.39-1.77)	1.45 (1.35-1.56)
20-24	1.23 (1.20-1.26)	1.29 (1.22-1.36)	1.21 (1.18-1.25)
25-29	reference	reference	ref.
30-34	0.92 (0.91-0.94)	0.96 (0.92-1.00)	0.91 (0.89-0.94)
35-39	0.89 (0.86-0.91)	0.96 (0.91-1.01)	0.87 (0.84-0.89)
40+	0.86 (0.83-0.90)	1.04 (0.96-1.13)	0.80 (0.76-0.85)
**BMI**			
15-<20	0.86 (0.83-0.89)	0.90 (0.83-0.97)	0.85 (0.81-0.88)
20-<22.5	reference	reference	ref.
22.5-<25	1.29 (1.26-1.33)	1.24 (1.17-1.31)	1.31 (1.27-1.35)
25-<27.5	1.68 (1.63-1.73)	1.49 (1.40-1.59)	1.74 (1.69-1.80)
27.5-<30	2.19 (2.12-2.27)	1.93 (1.80-2.07)	2.28 (2.19-2.37)
30-<35	2.98 (2.88-3.08)	2.51 (2.34-2.69)	3.14 (3.02-3.26)
35-<40	4.27 (4.07-4.49)	3.64 (3.29-4.03)	4.48 (4.24-4.74)
40+	5.26 (4.88-5.68)	4.96 (4.25-5.80)	5.37 (4.92-5.86)
**Origin**			
Nordic countries	reference	reference	reference
Rest of Europe	0.47 (0.45-0.49)	0.55 (0.51-0.60)	0.45 (0.43-0.47)
Rest of the world	0.45 (0.43-0.46)	0.65 (0.62-0.68)	0.39 (0.38-0.40)

**Smoker**	0.62 (0.60-0.63)	0.60 (0.57-0.63)	0.62 (0.61-0.64)
**In vitro fertilization**	0.85 (0.81-0.89)	0.92 (0.84-1.01)	0.83 (0.79-0.88)
**Nulliparity**	1.43 (1.40-1.46)	1.56 (1.50-1.62)	1.38 (1.35-1.41)
**Multifetal pregnancy**	2.76 (2.64-2.89)	6.11 (5.64-6.61)	1.77 (1.66-1.87)
**Comorbidities at baseline**			
Congenital heart disease	1.04 (0.93-1.16)	1.17 (0.94-1.45)	1.00 (0.87-1.13)
Valvular heart disease	1.32 (0.95-1.85)	1.92 (1.05-3.52)	1.13 (0.75-1.70)
Arrythmia	0.73 (0.61-0.88)	0.67 (0.47-0.97)	0.75 (0.62-0.91)
Hypertension	6.55 (5.87-7.29)	12.27 (10.13-14.86)	3.70 (3.29-4.16)
Heart failure	0.76 (0.46-1.24)	0.58 (0.25-1.37)	0.73 (0.43-1.24)
Myocarditis / pericarditis	0.84 (0.52-1.37)	1.45 (0.52-4.04)	0.74 (0.42-1.28)
Aortic disease / PAD	0.25 (0.08-0.73)	0.21 (0.03-1.49)	0.24 (0.06-0.92)
Myocardial infarction	0.62 (0.27-1.42)	0.98 (0.17-5.55)	0.58 (0.22-1.50)
Other coronary artery disease	0.51 (0.20-1.32)	0.88 (0.17-4.61)	0.39 (0.12-1.21)
Diabetes, type 1	5.52 (5.10-5.98)	11.07 (9.60-12.76)	3.98 (3.61-4.39)
Diabetes, type 2	1.48 (1.20-1.83)	1.73 (1.17-2.57)	1.39 (1.08-1.79)
Rheumatoid arthritis	1.36 (1.07-1.73)	1.92 (1.22-3.03)	1.22 (0.92-1.62)
SLE	2.35 (1.88-2.93)	5.38 (3.69-7.83)	1.50 (1.12-1.99)

The table shows odds ratios (OR) and adjusted odds ratios (aOR) after performing the generalized estimating equation. (PAD=peripheral artery disease. SLE=systemic lupus erythematosus.)
